# Comparison and Characterization of Android-Based Fall Detection Systems

**DOI:** 10.3390/s141018543

**Published:** 2014-10-08

**Authors:** Rafael Luque, Eduardo Casilari, María-José Morón, Gema Redondo

**Affiliations:** Universidad de Málaga, Departamento de Tecnología Electrónica, ETSI Telecomunicación, 29071 Málaga, Spain; E-Mails: rluque@uma.es (R.L.); mjmoron@uma.es (M.-J.M.); 061049251X@alu.uma.es (G.R.)

**Keywords:** fall detection, smartphone, eHealth, Android, accelerometer

## Abstract

Falls are a foremost source of injuries and hospitalization for seniors. The adoption of automatic fall detection mechanisms can noticeably reduce the response time of the medical staff or caregivers when a fall takes place. Smartphones are being increasingly proposed as wearable, cost-effective and not-intrusive systems for fall detection. The exploitation of smartphones' potential (and in particular, the Android Operating System) can benefit from the wide implantation, the growing computational capabilities and the diversity of communication interfaces and embedded sensors of these personal devices. After revising the state-of-the-art on this matter, this study develops an experimental testbed to assess the performance of different fall detection algorithms that ground their decisions on the analysis of the inertial data registered by the accelerometer of the smartphone. Results obtained in a real testbed with diverse individuals indicate that the accuracy of the accelerometry-based techniques to identify the falls depends strongly on the fall pattern. The performed tests also show the difficulty to set detection acceleration thresholds that allow achieving a good trade-off between false negatives (falls that remain unnoticed) and false positives (conventional movements that are erroneously classified as falls). In any case, the study of the evolution of the battery drain reveals that the extra power consumption introduced by the Android monitoring applications cannot be neglected when evaluating the autonomy and even the viability of fall detection systems.

## 1. Introduction

Owing to the socio-economic and health progress experienced by developed countries in the last 20 years, the older population has substantially increased, especially with the aging “baby boomers” (those born between 1946 and 1964). The remarkable growth of life expectancy has multiplied the number of senior citizens that face daily the risks of living on their own. Although it is well known that physical exercise avoids or delay the onset of diseases, it can also lead to falls, the major health hazard that diminishes the quality of life. Data from the World Health Organization [1,2], supported by different epidemiologic studies, indicate that a noticeable percentage of seniors aged over 64 (28%–35%) suffer a fall each year. This proportion increases to 32%–42% for those over 70 years of age. In fact, injuries caused by falls are one of the main causes of hospitalization for older persons, frequently resulting in a serious reduction of their independent living skills and even death. A fast reaction can remarkably diminish the effects of a fall on an older adult, but an immediate assistance is often not feasible if the injured individual lives alone and the injuries prevent the patient from seeking help. According to [3], the appraised fall incidence for independent living people over 75 exceeds 30% annually, as long as it has been estimated that up to 50% of nursing home residents suffer from falls every year (with more than 40% falling at least twice a year). In addition, up to 12% of all falls cause a fracture while 23% of trauma related-deaths in patients older than 65 (34% in those older than 85 years) follow a fall (see [4] for a state-of-the-art on this topic). However, physical damages associated to falls are not the only negative effect that must be considered. Fear Of Falling (FOF) has been recognized as a specific health problem, especially for older people. FOF, which is typically connected to an increase of neuroticism and anxiety, normally leads patients to strikingly reduce or evade physical activity. Thus, the psychological and emotional consequences of a fall contribute to degrade the independence of the elderly. Moreover, this loss of self-confidence deteriorates as older people age, leading them to a more acute social isolation and a lower quality of life.

This paper presents the prototype of an experimental system for fall monitoring. The prototype combines an Android-based smartphone as the platform hardware, a motion sensor (a built-in tri-axial accelerometer) and the location services supported by the smartphone. The election of a mobile phone-based system has evident advantages. On one hand, mobile phone-based applications can operate almost everywhere because of the popularity, decreasing costs and portability of mobile devices and the ubiquity of mobile technologies. In fact, the use of smartphones has grown to become a basic constituent of daily routine. Besides, most current smartphones seamlessly integrate all the required elements (accelerometers and gyroscopes) to develop autonomous and self-sufficient fall detection applications. An important point in the design of any healthcare monitoring application is ergonomics. Wireless communications clearly improve patients' mobility while the reutilization of an already existing personal device avoids the annoyances of carrying a separate fall detection gadget. Thus a smartphone oriented system does not introduce any specific attachable component in the life of older people, who in turn are becoming less reluctant to admit novel technologies to improve their safety and independence.

This paper is organized as it follows: after the introduction of this Section 1, Section 2 revises the related works. Section 3 presents the general structure and objectives of the developed system. Section 4 describes the design of the detection algorithms to be tested whereas the global system architecture and implementation are presented in Sections 5 and 6. In Section 7, the performance of the system and the accuracy of the acceleration-based detection algorithms are evaluated by means of extensive experiments performed on different scenarios. Finally, Section 8 draws the main conclusions of the work.

## 2. Related Work

Due to the advances in the area of electronic sensors and the widespread extension and cost reduction of personal devices, the research on systems for fall detection has rocketed during the last decade. Recent comprehensive works have thoroughly addressed the state-of-the-art on fall detection systems. In this sense, different criteria have been proposed to categorize the existing proposals. For example, the authors in [5] differentiate those studies that only take into account the recognition of the impact shock from those that also consider the “post-fall” phase. Alternatively, Perry classifies the detection techniques depending on whether the user's acceleration is measured and utilized to identify the fall [6]. The state-of-the-art presented in [7] also distinguishes between two general types of fall detection architectures: context-aware systems and those schemes that employ wearable devices with embedded accelerometers and, in some cases, gyroscopes aimed at sensing the user's position. As it refers to this latter type of fall detection systems with body-worn sensors, the FARSEEING project (funded by the European Commission) has supported a systematic bibliographic revision [8] of the related literature. In the conclusions, the study criticizes the lack of a methodological consensus to evaluate the proposed systems.

The recent study in [9] offers an interesting taxonomy of the systems and algorithms for fall detection. The performed classification considers three general categories: ambience device based, vision based and wearable device based detection systems. The ambient based approaches propose to combine audiovisual information and event sensing by capturing and analyzing floor vibrational data. The falls are tracked by means of pressure sensors in around the user. This may be a cost effective method but it leads to many false alarms due to spurious falls of other objects. On the other hand vision based assistive systems utilize cameras (or even microphones) to assess the user's behavior and detect events (as falls) without excessive intrusion in his/her routines. There are different strategies to perform the fall detection from the analysis of the video images, such us shape modelling using spatiotemporal features, study of the shape changes of the posture, 3D head position analysis, *etc*. For example, the systems by Anderson [10], Cucchiara [11], Diraco [12], Foroughi [13], Fu [14], Hazelhoff [15], Jansen [16], Lee [17], Liu [18], Miaou [19], Ni [20], Rougier [21], Sixsmith [22], Vishwakarma [23] or Yu [24] propose to detect the falls by processing the captured images of the monitored patient (or user). However this approach presents severe practical limitations. On one hand the required monitoring environment (which is normally limited to a closely observed room) is difficult to implement and expensive to maintain. Moreover, the quality of the images (and consequently, the accuracy of fall prediction) may be strongly determined by the illumination conditions of the room or by the existence of blind spots where the user cannot be properly monitored [25]. Apart from the vulnerability to noises from environmental objects [26], another problem of most existing vision based approaches is the absence of flexibility [9], as they are usually case specific and are designed and optimized for a very particular scenario. Besides, the individual's privacy is compromised, which directly translates into a lack of acceptance among users.

In any case, both ambient and vision (or image and audio processing) based schemes apply context aware techniques that require defining a supervision environment where the user activity is supposed to take place. In a similar way, the project described in [27] presents a fall detection architecture which is deployed through a network of non-invasive wearable sensing motes and an infrastructure of fixed motes that are conveniently distributed within the supervised monitoring scenario. Motes are equipped with low-power MEMS accelerometers so that when a fall is detected, the alert is forwarded to a base station via the fixed mote network. This networking infrastructure also allows the localization of the user. The detection algorithm is based on the angle of change, which is estimated from averaging the dot product of the acceleration vectors over 1 second.

Conversely, the approaches that employ wearable devices incorporate specific garments with embedded sensors (accelerometers) to estimate the motion (and, in some cases, the location) of the user's body in any unsupervised environment. If the wearable garments are also enabled with a wide area communication interface (e.g., 3G/4G data connections), the user can be ubiquitously monitored. Most smartphone-based fall detection systems can be clearly categorized into this family of “ubiquitous” detection techniques. As a main advantage, smartphone solutions do not constrain the user mobility to a particular monitoring zone while they provide a seamless inexpensive technology that is already integrated in the daily life of most potential users.

Nowadays, smartphones incorporate a wide set of embedded sensors, including not only accelerometers, but also cameras, microphones, digital compasses, gyroscopes or GPS units. The rapidly decreasing cost of the smartphones has fostered the adoption of this wearable technology and the measurement of the acceleration as the basis for fall detection. Consequently many smartphone-based architectures have been proposed over the last years.

Initial smartphone-based fall detection systems [28,29] were developed using the (today obsolete and discontinued) Symbian OS on Nokia phones. In this sense, Google's Android is the most dominant player in the smartphone industry, with 78.1 percent of the market share at the end of 2013 [30]. As a consequence, Android is selected as the Operating System (OS) and programming environment massively adopted by the literature on smartphone-based fall detection solutions. Conversely, there is much less literature devoted to fall detection architectures deployed on other operating systems for mobile devices, such as iOS (as the systems presented in [31,32], or [33], where an iPhone is in charge of receiving and processing the signals from diverse external mobility sensors to warn the user about potential falls or to estimate the fall risk for post stroke patients). In [34] (a work of 2011) a generic fall detection Java multiplatform software architecture (using an external accelerometer) is implemented in both a Symbian phone (Nokia 5800) and Android smartphones (Samsung Galaxy, HTC Hero). However, the system is not tested and no comparison between the performances of these two OS is offered. On the other hand, authors in [35] discuss the capabilities of different mobile operating systems (Windows Phone, Meego Harmattan, Symbian and Android) to develop applications for fall detection. Authors conclude that the best option is Android as it provides more support from APIs and reduces the implementation time.

PerFallD is the Android architecture developed by the authors of the theoretical study in [36,37]. The system, which is founded on the technique of the acceleration thresholds, has served as a one of the references for the implementation of the fall detection algorithms tested in this article. As later discussed, the detection decision of PerFallD is carried out considering the values of the total acceleration of the phone body and the absolute vertical acceleration during a certain observation time window. A very similar procedure is followed by the systems presented in [38,39]. In the second case, the platform also informs about the direction of the fall. In order to detect free falls, authors in [40] suggest measuring the net displacement of the user for the time during which the acceleration is close to zero. Although the algorithm is intended for Android-enabled devices, the detection technique is not implemented nor tested in an actual smartphone.

In order to minimize the requirements of computational power and real time processing in the smartphone, other systems, such as that proposed in [41], just consider a simple single-threshold algorithm to deploy the fall detection. In another work of the same authors [42], an impact is presumed if the acceleration is greater than an empirically adjusted threshold of 2.3 g. An impact is only considered to be a fall if the final user orientation is horizontal.

Another Android App for smartphone-based fall detection is iFall (Android Application for Fall Monitoring and Response) [43], which is available at Google Play Store. The theoretical basis for the employed detection algorithm is similar to those of PerFallD, as the utilized algorithm is also grounded on the same acceleration threshold technique. Unlike PerFallD, the fall detection criterion considered by iFall uniquely depends on the global acceleration.

The work in [44] combines a smart sensor and camera-enabled smartphone to detect and check the occurrence of a fall. As soon as the sensor detects a fall event, live data are streamed to a remote monitoring point while the patient is personally attempted to be contacted to get a vocal or keypad feedback. In [45] an Android smartphone with 3-axial accelerometer is again considered as a telehealth device. The connection to the remote telemonitoring unit is accomplished by means of a TCP/IP socket via Wi-Fi. The paper in [46] presents another smartphone-based system that tracks the user's movements and automatically transmits an alarm message to the caregivers whenever a fall is recognized. A multilayer perceptron (*i.e.*, a neural network) is utilized to analyze the user's mobility pattern.

Viet [47] jointly considers the information from the orientation sensor and the accelerometer of a smartphone to detect the falls. The authors state that the posture of the user before the fall must be taken into account to optimize the threshold that determines a detection. The election and optimization of the decision thresholds and the observation time window in accelerometer-based Android programs are still open issues. The Android program portrayed in [48] parameterizes these values taking into account the age, sex and Body Mass Index of the user to be monitored.

The authors in [49] introduce a system that makes use of a smartphone with an embedded tri-accelerometer mounted on the waist. By analyzing the data from the accelerometer, the smartphone obtains the information about the user's motion, which is categorized according to five different patterns. A fall is assumed to occur when the Signal Magnitude Area (calculated from the integration of the accelerometer signals), the acceleration magnitude vector and the tilt angle simultaneously exceed the corresponding thresholds. In that case, a Multimedia Messaging Service (MMS) with GPS coordinates is sent to the remote monitoring point.

The study in [50] evaluates the specificity and sensitivity of the smartphones to detect falls when their performance is compared to that achieved with independent accelerometers. Results show that smartphones are a valid option for detecting falls with high accuracy.

Most proposed smartphone-based solutions are “smartphone-only” architectures where no supplementary components (apart from the phone's built-in sensors) are utilized [51]. The incorporation of additional high resolution sensors in the system may introduce a higher accuracy in the measurements while avoiding the need of wearing the smartphone in a fixed position (where the detection process is assumed to be optimal). On the other hand, extra elements increase the cost of the system hardware as well as it may reduce the perceived usability of the fall detector (as more devices must be worn by the user). For example, the system presented in [52] combines a smart phone and a digital watch with wireless communications (the EZ430-Chronos model provided by Texas Instruments, which integrates a three-axis accelerometer) to detect the falling and to get in touch with the emergency contacts.

The platform described in [53] also integrates an extra electronic device (a SensorTag from Texas Instruments) to monitor the user's movements. The accelerometry data are processed in the SensorTag, so if a fall is detected, a message is sent via Bluetooth to the smartphone (which just acts as a communication gateway between the external sensing device and the remote monitoring point). Paper in [54] implements a fall detection system with an Android-based watch equipped with a tri-axial gravity accelerometer. The watch does not incorporate any Wide Area Network communication interface. Thus, upon detection of a fall, the device just issues a vibratory alarm.

As it can be deduced from the aforementioned studies, accelerometry is, by far, the most extensively employed method for the detection of falls in smartphones. The position where the accelerometer is located (waist, wrist, thigh or chest) has been used as a criterion to classify the existing solutions based on tri-axial accelerometers [55]. In some systems, the information obtained from the accelerometers is combined with data from inertial sensors or gyroscopes.

There are two general strategies to detect the fall occurrences from the data obtained by the accelerometers:

- Classification of the movement founded on Pattern Recognition Methods (PRM) that employ data bases, training phases and AI (Artificial Intelligence) solutions. In the systems proposed by Ganti [56] and Karantonis [57], in order to avoid false alarms, the activity patterns of the patient are characterized. For that purpose, these authors propose a complex “training” phase where the values measured by the mobility sensors when the user performs daily activities of diverse nature are stored in a database. Once this characterization is finished, these values previously captured are utilized to distinguish the movements of a normal situation from an alert condition. Authors in [58] employ a waist worn fall detection system to compare different machine learning classification algorithms to detect falling patterns. The study concludes that multilayer perceptrons perform better than other classification techniques. This type of strategies permits tuning the detection algorithm for the particular behavior of the user. Conversely, the need of incorporating training phases, databases and/or AI techniques hinders their implementation in a hardware and battery limited multifunctional device like a smartphone.
- Detection based on acceleration thresholds (Threshold Based Detection or TBD): In [59] Nyan utilizes an acceleration threshold that is based on the absolute peak values of the accelerometer's measurements. In the experiment, the accelerometer is transported in a garment near the shoulder. Kangas [60,61] proposes four thresholds for the following magnitudes: the vertical acceleration, the total acceleration vector, the dynamic acceleration vector and the difference between the maximum and minimum modules of the acceleration. A fall is assumed whenever one of these thresholds is crossed. The results of the performed tests show that a simple triaxial accelerometer attached to the waist or head can be accurate enough to detect most falls, even with quite simple “thresholding” algorithms. Before the apparition of the smartphones, the detecting devices that were employed by these algorithms required a specific and, in some cases, scarcely portable hardware. However, as smartphone natively integrates accelerometers and gyroscopes, methods based on acceleration- thresholds provide a good trade-off between the results and the complexity of the algorithm's implementation.

Table 1 offers a general review of the literature on Android-based systems intended for fall detection or fall prediction. For this purpose an extensive bibliographic revision was performed and 56 articles (from 2009 to May 2014) proposing Android fall detectors were found. The table classifies the proposals according to different criteria, summarizing the main characteristics of the fall detectors.

The first classification distinguishes the general topology of the system: body-worn or context-aware systems. As the table shows, the majority of the architectures can be categorized as body-worn systems, *i.e.*, body area networks that include an Android-enabled device (normally a smartphone). Nevertheless there exist also examples where the Android device is the center of a context aware system (e.g., an embedded computer installed on a wall in [62], which detects the falls by means of a Doppler sensor). Similarly there are also architectures [63–65] that combine context aware techniques and body-worn devices. For instance, in [63] authors propose to combine the data of video cameras and the accelerometer of a wearable Android platform to characterize the user mobility and detect falls.

An important aspect in the architecture of the detection system is the role of the Android device. Table 1 informs if the Android device is employed as a Sensor (S), as a Data Analyzer (DA) to decide if a fall has occurred, as a Communication Gateway (CG) to retransmit the sensed data (or the fall detection decision) to a remote server, or/and just as Remote Monitoring Unit (RMU) offering an interface to warn about the falls. As it can be examined in the table, most solutions benefit from the sensing, computing and communication capabilities of smartphones, which can implement and execute the fall detection algorithm while simultaneously acting as a sensor and as a data gateway. In this sense, the table also differentiates those systems that concentrate all the functionalities on a single smartphone (smartphone-only or SP-only systems) from those which combine a smartphone and one or several external wireless sensors. On the other hand, there are a few examples of systems that do not consider the use of a smartphone and are deployed on Specific Devices (SD) (a hardware platform purposely designed for fall detection). In this category we could mention the work in [41] where an Android-based watch is utilized as the mobility sensor of the system.

The sensor (or sensors) that the revised systems employ is indicated in a particular column of Table 1. The measurements from the built-in tri-axial accelerometers of the smartphones are by far the most utilized magnitudes to evaluate the possibility of a fall occurrence. Just in some cases, embedded smartphone gyroscopes or external accelerometers are considered. The small range and precision of built-in accelerometers have been stated as inappropriate to detect falls [66]. However, authors in [67] found that the use of dedicated accelerometers to detect falls presents similar results to those obtained with smartphones.

Finally, the fall decision algorithm that is utilized by the system is shown in the last column of the table. The table reveals that simple Threshold-Based Detection (TBD) systems are preferred to complex Pattern Recognition Methods (PRM), which usually impose higher computing and memory costs as well as long training phases to adapt the algorithm to the particular characteristics of the user to be monitored.

Just a few of the aforementioned proposed Android systems (such as iFall [43] or that by Kerdegari [58]) have been released to the general public. Other available apps (Spantec Fall Detector, Fall Monitor, T3LAB, Fade Fall Detector, *etc.*) do not provide any detailed information about the detection algorithm employed or any insight about the performance achieved by the software. As a matter of fact there is no consolidated Android-based product in this realm (see Google Play Store [68] for more details about these applications).

As it refers to commercial systems for fall detection, most existing professional solutions are normally equipped with a specific attachable hardware which is in charge of measuring the user's motion. We can mention the following products:

‐ Brickhouse [69] provides a typical system with two functional components: a portable sensor (which is attached to the belt and placed on the user's waist) to detect the movements, and a fixed gateway connected to the wired phone line. The gateway is in charge of receiving the signals from the sensor and communicating any eventual emergency situation to the medical staff. Obviously, this high costly system can only operate in a home supervised environment where the distance between the sensor and the gateway is below a maximum value in order to guarantee the viability of the connection. Besides, the employed algorithm to detect the falls is not described.
‐ Betterbuys [70] is an economic system which is deployed through a set of sensors located in cushions at those household locations commonly frequented by the user (on the chairs, beds, floor mats, *etc.*). The device is equipped with a volume control and an interphone so that, in case of emergency, it emits a musical tone and a flashing LED-type light signal to alert anyone nearby. However, as in the previous example, this system has evident limitations to achieve a persistent ubiquitous fall detection.
‐ ITT EasyLifeS [71] is another type of system that integrates the two components (sensor and communication unit) in the same terminal. It consists of a mobile phone equipped with a balance sensor. The manufacturers claim that when the phone is dropped, it automatically proceeds to dial the corresponding emergency phone number. Its main drawbacks are twofold: Firstly it employs an unconventional device, and secondly, the election of the triggering thresholds is too simple to ensure an accurate detection.
‐ Philips Lifeline [104] consists of a device (to be hung on the neck) that incorporates a “panic button”. When the button is pressed, a warning message is reported to the appropriate medical staff. Nowadays this type of service based on alarm buttons is quite widespread among older people and it provides a very simple and effective solution to deal with falls. Though, it cannot handle certain critical situations such as a sudden collapse that causes loss of consciousness or cases with handicapped patients that are not able to push a button.

Despite the efforts to achieve a reliable system, all the aforementioned commercial products reveal some kind of deficiency that hinders the efficiency and ubiquity of the fall detection. Firstly, they present a higher cost than smartphone-based solutions. Besides they oblige to wear an additional and specific garment, while communications are normally limited to indoor scenarios and short range transmissions as the use of a base-station is required in many cases. Furthermore, they offer closed solutions that cannot be easily reprogrammed, customized or adapted to the particular conditions of the user or the application. In addition, the typical algorithms employed for the detection cannot be numerically parameterized (e.g., the decision thresholds). In fact, the utilized detection algorithms are not usually described by the vendor in the documentation of the product.

## 3. Structure of the Prototype and Objectives

The main purpose has been to apply mobile technology in health care field by developing and implementing an Android platform-based prototype system, named Monitoring Elderly People with Dementia (MonEPDem) to monitor older people with early dementia. The system is operative for both indoor and outdoor environments and it requires no extra hardware or service cost (apart from those derived from the use of a smartphone).

**Figure 1.**
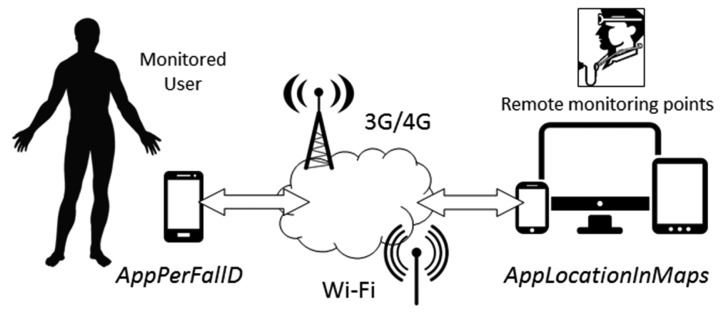
MonEPDem System architecture.

The prototype, which is sketched in Figure 1, consists of two applications called, respectively, Application for Pervasive Fall Detection (AppPerFallD) and Application to Display Location In Maps (AppLocationInMaps). AppPerFallD is conceived to be executed on the smartphones of the monitored individuals. In case of a hypothetical fall, the application provides the required tools to detect and report the information about the incidence to a remote monitoring point, allowing a quick assistance in the event of a serious injury. The alarm is transmitted by means of a vocal conversation or a text message (a SMS) containing the GPS coordinates, either over the mobile phone network (3G) or over Wireless Fidelity (Wi-Fi). Simultaneously, for every detected fall, AppPerFallD also stores the coordinates and a timestamp in the smartphone by means of a SQLite database. In order to perform these tasks properly, the application is required to manage accelerometer events in an efficient way, real-time positioning mechanisms, SQLite Databases and, naturally, the communication interface to use.

Aiming at enabling an efficient and pervasive fall detection, AppPerFallD allows selecting different fall detection algorithms that utilize acceleration threshold-based techniques, which can benefit from the built-in sensors (accelerometer and gyroscope) which are commonly integrated in most present smartphones. In this paper, we utilize the deployed platform to compare the performance of these algorithms.

Besides, AppLocationInMaps, which runs on the remote monitoring point (also a smartphone), is continuously ready to receive alarm SMS messages. In case of a fall, this Android app displays the most recent (or the last known) location of the monitored user, by plotting it on a map downloaded from the Google Maps web service [105].

## 4. Fall Detection Algorithms

Our goal is to implement and compare different acceleration-based fall detection techniques proposed by the literature. In particular we consider the following algorithms:

### 4.1. Basic Monitoring of the Acceleration

For the fall detection this basic algorithm only uses the module (|*A_T_*|) of the total acceleration of the phone (*A⃗_T_*). This module can be computed as:

(1) $$\left|A_{T}\right|=\sqrt{{\left|A_{x}\right|}^{2}+{\left|A_{y}\right|}^{2}+{\left|A_{z}\right|}^{2}}{\left(m/s\right)}^{2}$$

where *A_x_, A_y_* and *A_z_* are the acceleration readings in directions of *x, y*, and *z*-axis measured by the accelerometer that is embedded in the smartphone.

A fall is directly assumed if the measured module of the acceleration exceeds a decision threshold. Thus, the detection decision only considers brusque peaks in the acceleration, neglecting the analysis of the complex behavior of the acceleration vector whenever a fall takes place. As a consequence, this algorithm is prone to the detection of false positives (*i.e.*, the identification of any type of sudden movements as fall occurrences).

### 4.2. Fall Index

A *Fall Index* (FI) is suggested by Yoshida [106]. For the *i*-th sample of the acceleration, *FI* can be computed as a function of the 20 last measurements of the acceleration in the *x, y*, and *z*-axis (*A_x_, A_y_*, and *A_z_* respectively):

(2) $$FI_{i}=\sqrt{\sum_{k=x,y,z}\sum_{i-19}^{i}{\left({\left(A_{k}\right)}_{i}-{\left(A_{k}\right)}_{i-1}\right)}^{2}}$$

A high sampling frequency of the acceleration vector is normally established for a proper computation of FI in the case of sudden falls. However, according to this strategy, most falls that occur slowly (*i.e.*, without sudden variations of the acceleration) may go unnoticed.

### 4.3. PerFallD

PerFallD [37] algorithm simultaneously takes into account the values of the modules of the total acceleration of the phone (*A⃗_T_*) and the acceleration at the absolute vertical direction (*A⃗_V_*), which can be estimated as:

(3) $$\left|A_{v}\right|=\left|A_{x}\sin\theta_{z}+A_{y}\sin\theta_{y}-A_{z}\cos\theta_{y}\cos\theta_{z}\right|$$

where *θy* and *θz* are the measured pitch and roll values, which determine the mobile phone's orientation. These angles are sensed by the gyroscope integrated in the smartphone.

The algorithm separately analyses |*A_T_*| and |*A_V_*|. Thus, in order to assess the occurrence of a fall, the algorithm considers two phases for both parameters.

If the difference of the estimated value of |*A_T_*| within an observation time window (*win_tt_*) surpasses a certain triggering threshold (*Th_tt_*), the pattern recognition phase is initiated. During this second phase the difference between the maximum value and the minimum value of |*A_T_*| is computed within a second checking time window (*win_ct_*) after *win_tt_*. If this difference does not exceed another threshold (*Th_ct_*), a possible fall is considered to be detected. A similar process is applied to |*A_v_*|, with the corresponding time windows *win_tv_* and *win_cv_* and the thresholds *Th_tv_* and *Th_cv_*. A fall is only assumed to have occurred if both detection conditions about |*A_T_*| and |*A_v_*| are satisfied.

### 4.4. iFall

This algorithm [43] takes into consideration that a fall initially provokes a sudden and significant decrease in the acceleration amplitude. After this “free-fall-period”, the acceleration experiences an abrupt spike as soon as the body hits the floor. Consequently, if the acceleration |*A_T_*| crosses a lower and an upper threshold during a certain observation time window, a fall is suspected. However, the fall is only reported if the patient really begins from an upright position and ends in a horizontal position. For that purpose, if the vertical position is restored (or if a dropped smartphone is picked up) within a “post-fall” observation period, the detection event is neglected. Otherwise, if the vertical position is not recovered before this time expires, the system assumes that the patient is lying on the ground and the alarm is emitted.

## 5. System Design

**Figure 2.**
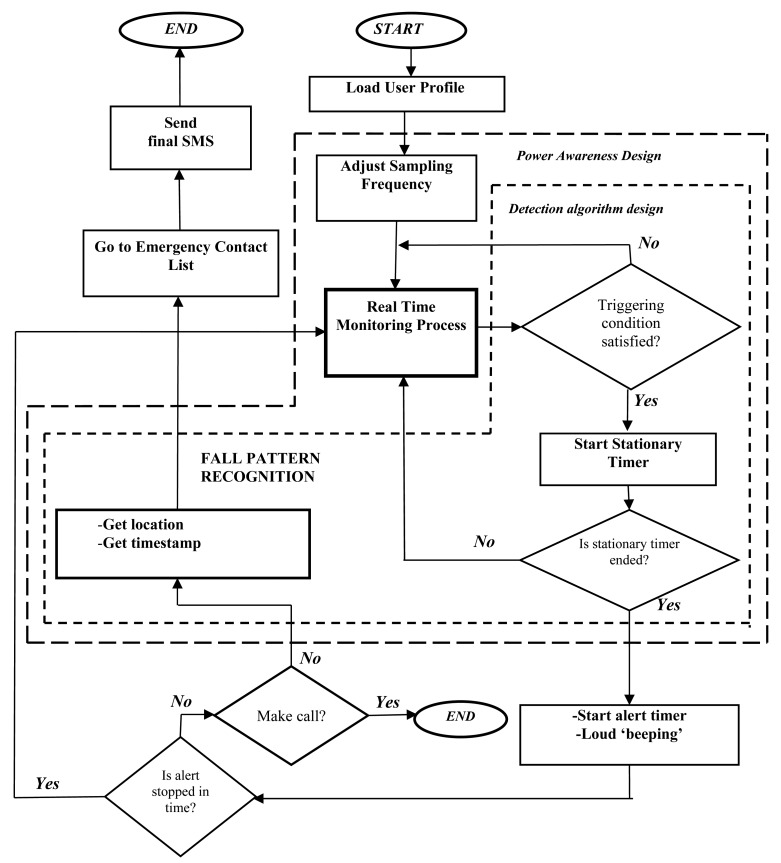
Working procedure of the system.

The general workflow of the developed program is illustrated in Figure 2. As soon as the program is started, a user profile is loaded containing the configuration of the fall detection system (selected detection algorithm, sampling frequency of the accelerometer, thresholds, *etc.*) and the personal data and preference of the user (e.g., an emergency contact list, alarm tone, *etc.*). These parameters of the profile are fully configurable by the user.

After the program is parameterized, the monitoring process is launched. Thus, the real-time data collected by the accelerometers are permanently compared to the detection thresholds according to the selected algorithm (the modules within the smallest dashed box of the figure are executed). If the preset thresholds are surpassed (a fall is presumed because of a certain value or values of the acceleration), a “stationary phase” is initiated to confirm that a fall may have occurred. This phase corresponds to the “pattern recognition phase” and the “post-fall” observation period of the PerFallD and the iFall algorithms, respectively. On the contrary, the duration of this phase is set to 0 if these algorithms are not considered.

When the stationary phase concludes (and the fall is confirmed), another timer is executed. During this new period, the smartphone emits an acoustic alarm to inform the user that a fall has been detected. If no response from the user is received through this time (a screen button is not touched), a fall is assumed and the alert notification is triggered. In this case, the application obtains the GPS coordinates of the user and a timestamp. Depending on the configuration of the program, this information can be directly sent by a text message to a set of predefined contacts (selected by the user in the emergency contact list) or, otherwise, the application can make a phone call to a certain number also specified in the configuration. On the other hand, if the acoustic alarm is turned off before the corresponding timer elapses, the application returns to the normal monitoring process.

## 6. System Implementation

The prototype is initially developed on a HTC Desire X smartphone. This device features an ARM-based architecture, dual-core CPU working at 1 GHz, with 768 MB RAM memory, a 10.16-cm screen, GPS sensor and 4 GB of internal storage. It is powered by a 1650 mAh rechargeable lithium ion battery and incorporates an embedded accelerometer/G-sensor. The OS version employed by the phone is Android 4.0. The system is put into operation by the two abovementioned software applications: AppPerFallD, which implements the detection algorithms, and AppLocationInMaps (in the remote monitoring point). Both programs are implemented in Java, with Eclipse and Android Development Tools (ADT) plugin. The system was also installed and tested on an HTC Sensation XE model, provided with similar sensors, a 10.922-cm screen and Android 2.3.4 Gingerbread OS.

**Figure 3.**
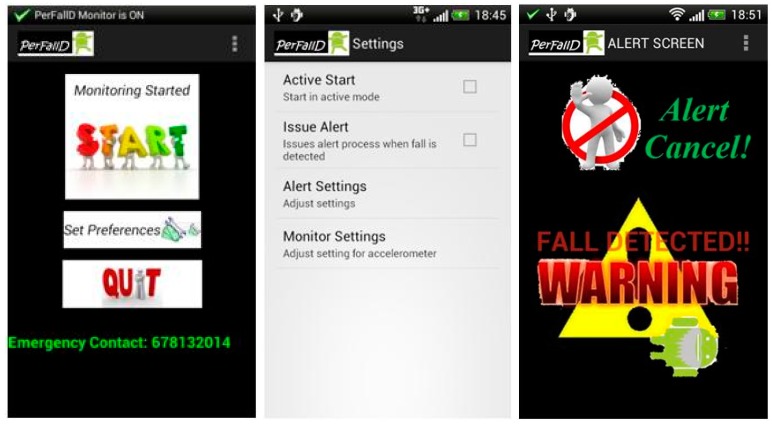
Snapshots of the User Interface (UI).

The two main software modules of AppPerFallD are:

- MonitoringPerFallD: It includes a UI (User Interface), which is designed for older people by following the elderly-friendly design ideas from Jitterbug [107]. Thus, in order to ease its use, the UI incorporates a reduced set of lit key buttons with clear options and no confusing menus. Three screenshots of this user interface are illustrated in Figure 3.
- Detection Service: It is the monitoring service that implements the fall detection algorithms. To execute these algorithms, the service is in charge of collecting and recording the readings of the sensors. These readings are processed basing on a power-aware strategy.
- Other four specific modules handle the rest of the functionalities of the application: the transmission of fall alerts, the smartphone connectivity (via Wi-Fi or UMTS), the location processing and the management of a SQLite Database to store the monitored user location.

Additionally, AppLocationInMaps is the application developed for the remote monitoring point. Its goal is to receive, decode and present the information contained in the alert messages that AppPerFallD transmits when a fall is detected. Among other functions, the application displays the patient's location on a map downloaded from Google Maps web service.

## 7. Evaluation of the System and Detection Algorithms

The analysis focuses on the performance of the implemented fall detection algorithms as well as on the resource consumption of the application.

The algorithms were evaluated by a series of methodical experiments. Thus, a set of different movement patterns (including falls) are simulated by 15 different volunteers (six females and nine males, aged between 15 and 68 years and 150–190 cm tall with an average weight of 70 kg) in an indoor environment (a domestic living room). The subjects emulated the falls according to three directions (forward, lateral and backward), at different speeds and over a pad to reduce the impact. The rest of simulated movements consist of diverse “Activities of Daily Living” (ADL) such as jogging, walking, standing, sitting or answering the phone. Experiments were repeated by changing the position where participants placed the smartphone: attached to the waist (by means of a belt) or next to the thigh (within a trouser pocket). Each individual carried out more than 50 movements (comprising at least 25 simulated falls and 25 simulated ADLs) for every algorithm and every position under test.

In order to evaluate the ability of the algorithms to discriminate the fall detection patterns, we computed the number of false negatives (*i.e.*, those falls that remained undetected) and false positives (*i.e.*, those ADL movements that were incorrectly identified as falls and provoked the transmission of an alert). The estimation of the false positives does not take into consideration the possibility that the user can cancel the alerting process after a fall is detected and the local acoustic alarm is triggered in the smartphone (that is to say: user-cancelled alerts are also computed as false positives). The selected thresholds for the algorithms were also the same selected in the tests investigated in [37].

For comparison purposes, we set all the thresholds and time windows of the algorithms to the same values utilized in the bibliography [37,43,106]. For the *PerFallD* algorithm we employed *Th_tt_* = 150, *Th_tv_* = 6, *Th_ct_* = 50, *Th_tt_* = 2, *win_tt_* = *win_ct_* = *win_tv_* = *win_cv_* = 4 s. For iFall, we set the lower and the upper thresholds to 1G and 3.5G respectively (this upper thresholding limit is also chosen for the basic algorithm and for the Fall Index algorithms). These settings are selected basing on training data and aiming at minimizing the false negatives while reducing the false positives to a reasonable minimum.

Table 2 presents the percentages of false negatives (ratio between the number of false negatives and the number of simulated falls) and false positives (ratio between the number of false positives and the number of ADL movements) measured when the different algorithms are employed and the smartphone is attached to the waist. For comparison purposes, the table also incorporates the results obtained with a commercial specific device for fall detection in a very similar test scenario in [37].

These results show that PerFallD and iFall algorithms offer better results than the basic “thresholding” methods (such as the basic monitoring of the acceleration and the algorithm that is presumed to be used in the commercial product). We think that this is due to the fact that PerFallD and iFall algorithms assume a more complex and realistic fall pattern with at least two phases and a certain “observation window”. This observation window is also defined by Fall Index (as long as it takes into account the evolution of last 20 samples of the acceleration components). In fact, the Fall Index algorithm exhibits relatively good results just basing its detection decision on the evolution of the changes in the global acceleration during a small time interval. The algorithms that incorporate a longer analysis of the user activity before a fall is assumed also reveal a more homogeneous behavior when the fall pattern (*i.e.*, the fall direction) is modified. In this case, results (as those obtained by the commercial device) suggest that the typology of the tested falls is a key aspect when assessing the capability of the system to detect the fall event. In any case the benefits of using certain algorithms are not as evident as those reported in other studies, such as [37].

For the case of PerFallD algorithm, Table 3 includes the comparison of the measurements when the position of the smartphone is varied. Except for the case of forward falls, these tests indicate that a better performance is achieved if the smartphone is attached to the waist. This can be explained by the fact that the tracking of a point next to the waist can better reflect the movement of the center of mass of the body [108]. These results are coherent with the conclusions of the study in [61], which compared the performance of accelerometer-based fall detection systems when the accelerometer (not in a smartphone) was alternatively located on the wrist, the waist or the head. In contrast with those studies that recommend attaching the smartphone to the chest [38], ergonomically the placement of the detection device by the waist also introduces less restriction on body movement and reduces the user's discomfort [50]. Moreover, waist belts are normally not considered as invasive by the older people [41].

**Figure 4.**
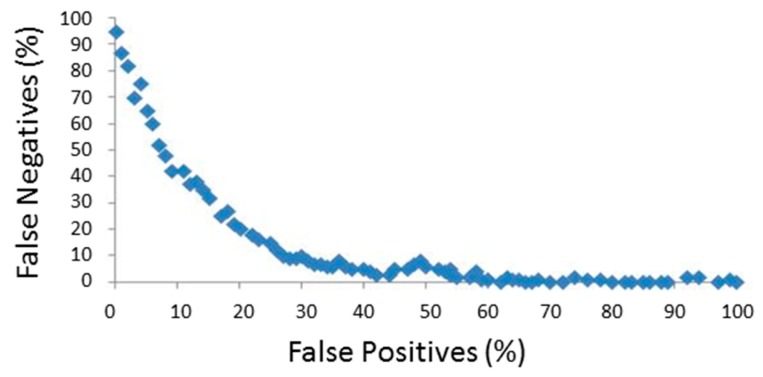
Relationship between the percentages of False Negatives (FN) and False Positives (FP) for different values of the detection threshold *Th_tt_* when *Th_ct_* is set to a fixed value. Each point in the graph corresponds to the utilization of a different value of the *Th_tt_* threshold.

An important point in the study of acceleration-based techniques is a proper selection of the detection thresholds. In most studies, the values for these thresholds are heuristically selected. In this sense, a trade-off to simultaneously avoid false positives (FN) and false negatives (FP) must be achieved. To illustrate the importance of this trade-off, the scatter plot in Figure 4 shows the percentages of false negatives and false positives (for the PerFallD algorithm) when one of the employed threshold is modified. These experimental Receiver Operating Characteristic (ROC) type-curves can be employed to set threshold values that guarantee the compromise between low FN and FP percentages.

### Analysis of the Power Consumption

A crucial aspect when evaluating a smartphone oriented software is power consumption. Complex computation or massive operation of the sensors may cause heavy battery consumption and make monitoring applications virtually infeasible from a practical point of view.

In general, power drain in smartphones is highly dependent on several features such as electrical and network setting, user location, signal power, user activity, phone utilization, *etc*. To isolate the impact of the fall detection app on the smartphone power consumption, we perform a series of tests in which we compare the battery discharge for different activity conditions of the fall detection system.

In particular, for each test, the phone battery is fully charged and, after that, the power state is periodically monitored. No other additional application was executed in the phones during the experiments. Three diverse scenarios are considered:

- Scenario 1: AppPerFallD runs in passive mode without executing the detection algorithms. Consequently, the mobility of the smartphone does not affect the consumption.
- Scenario 2: AppPerFallD runs in active mode, *i.e.*, the fall detection algorithms (in this case PerFallD) are activated. This implies that the acceleration values measured by the G-sensor are continuously processed. In this case the smartphone is kept in a completely static position.
- Scenario 3: The application is also active, but, in this case, the smartphone undergoes a pattern of periodical simulated falls. Consequently, the corresponding alerting SMS messages are transmitted to the remote monitoring point. These SMSs inform about the position of the user. Thus, the GPS coordinates need to be obtained.

**Figure 5.**
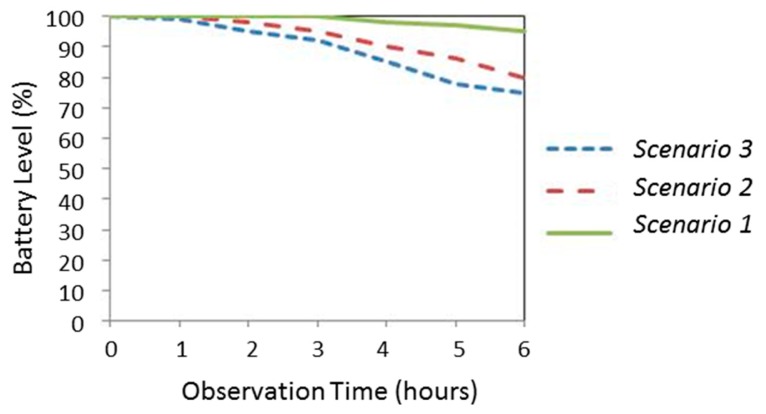
Estimation of energy consumption in the HTC Desire X phone.

**Figure 6.**
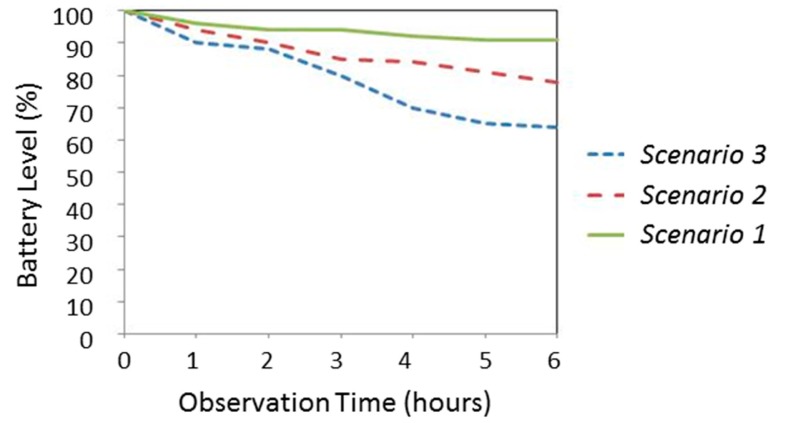
Estimation of energy consumption in the HTC Sensation XE phone.

Figures 5 and 6 show the evolution of the battery consumption for the three scenarios when the monitoring application is running during 6 h on the HTC Desire X and HTC sensation XE models, respectively. For the measurements, the battery state was obtained by the Diagnosis-System Information application provided by the Android Operating System.

For both smartphone models, results indicate that the monitoring application has a not negligible repercussion on power consumption (in both cases, the battery was exhausted before 40 h under the conditions of the scenario 2). The graph for the scenario 3 evidences that consumption can severely increase if the application utilizes updated information from the GPS.

## 8. Conclusions

Smartphone-based architectures for pervasive fall detection can clearly benefit from the massive social acceptation and widespread extension of smartphones. These devices, which natively integrate accelerometers, gyroscopes and diverse communication interfaces (Wi-Fi, Bluetooth, 3G and beyond data connections), provide a cost-effective and efficient solution for the deployment of wearable systems for fall detection and alerting.

This paper has presented a prototype of a fall detection system based on Android applications for mobile phone platforms. The system is in charge of sending a message or automatically establishing a phone call whenever a fall is presumed.

Most works in the literature about smartphone-based fall detection architectures base the identification of fall patterns on the analysis of the data reported by the built-in smartphone accelerometer (in some cases, combined with the information of the phone orientation). Although there are solutions that employ trained AI systems to discriminate the falls from the conventional physical activity of the users, the hardware limitations of the memory and real-time processing capabilities of the smartphones recommend implementing less sophisticated detection procedures. In this sense, the majority of the proposals apply simple threshold-based techniques to process the sequence of acceleration data. In this work, the developed prototype, tested with different smartphone models, was aimed at evaluating different existing algorithms that utilize threshold comparison methods to identify the falls. For this goal, a wide set of experiments executed by 15 volunteers were conducted. Experiments included a mixture of simulated falls and conventional movements. In contrast with the conclusions of other studies (where a new algorithm is proposed), the obtained results reflect the difficulty of determining an optimal strategy to detect falls. For example, no algorithm achieves an efficiency higher than 95% or 90% to avoid false positives and false negatives, respectively. In an actual application environment, this could imply that many falls could be unnoticed while many movements related to regular activities could provoke alarms that should be manually deactivated by the user before an alert message is sent to a remote monitoring point. The strong dependence of the measured performance on the typology (*i.e.*, direction) of the falls indicates that any fall detection system must be evaluated through an exhaustive test-plan with a high diversity of movement patterns. The study of the trade-off between “false positives” and “false negatives” also reveals the importance of the selected thresholds, which completely govern the accuracy of the detection process. In addition, the limitations introduced by the battery lifetime may become a remarkable element to determine the viability of this type of fall detection systems in a real application environment where a user should be permanently (24 h a day) telemonitored. The constant use of the accelerometer and (if needed) the GPS sensor by the detection algorithms undoubtedly reduces the autonomy and applicability of smartphone-based architectures (in our experiments, less than 40 h of continuous monitoring were accomplished). Consequently fall detection Android applications must be carefully designed to optimize the access to the employed sensors and to minimize power consumption.

Ergonomics and usability are other two key aspects for the actual adoption of this type of technology (especially among the older people, who are the main target of these systems). In this sense, the need for frequent interaction of a not-expert user (battery charging, cancellation of false alarms, programming of detection thresholds, complex training phases to characterize the user's activity patterns, *etc.*) may noticeably hinder the acceptance of these telemonitoring services.

## Figures and Tables

**Table 1.** Classification and main characteristics of the Android-based solutions for fall detection systems.

**Paper**	**Year**	**GENERAL TIPOLOGY**	**ROLE OF THE ANDROID DEVICE (Possible Role(s) of the Android-Enabled Device:** ‐ **Sensor (S),** ‐ **Data Analyzer for Fall Detection (DA),** ‐ **Communication Gateway (CG)** ‐ **Remote Monitoring Unit (RMU)**	**COMPONENTS: SP-Only, SP Combined with External Sensors, SD (Specific Device)**	**EMPLOYED SENSORS**	**FALL DECISION ALGORITHM** ‐ **Threshold-Based Detection (TBD)** ‐ **Pattern Recognition Methods (PRM)**
[43]	2009	Body Worn	S, DA, CG	SP-only	Built-in tri-axial accelerometer	TBD

[36,37]	2010	Body Worn	S, DA, CG	Combined (SP and an external magnet)	Built-in accelerometer (in [37] also a magnetic sensor)	TBD

[72]	2010	Body Worn	S, DA, CG	SP-only	Built-in tri-axial accelerometer	TBD

[73]	2010	Body Worn	S, DA, CG	SP-only	built-in tri-axial accelerometer and magnetometer	PRM

[74]	2011	Body Worn	S, DA, CG	SP-only	Built-in tri-axial accelerometer	A combination of TBD and PRM (state machine-based)

[50]	2011	Body Worn	S, DA, CG	SP-only	Built-in tri-axial Bosch Sensortec's 3-axis	TBD
					BMA150 accelerometer	

[75]	2011	Body Worn	CG	Combined	Specific Android based Personal Activity Monitor with accelerometer	TBD

[76]	2011	Body Worn	S, DA, CG	SP-only	Built-in accelerometer	TBD

[41]	2011	Body Worn	S, DA, CG	SP-only	Built in accelerometer and orientation sensor	TBD

[77]	2011	Body Worn	S, DA, CG	SP-only	Built-in tri-axial accelerometer	TBD

[47,78]	2011	Body Worn	S, DA	SP-only	Built-in tri-axial accelerometer	TBD
	2012					

[79]	2012	Body Worn	S, DA, CG	SP-only	Built-in tri-axial accelerometer	PRM: finite state machine

[80]	2012	Body Worn	S, DA	SP-only	Built-in tri-axial accelerometer	PRM: self organizing map

[81]	2012	Body Worn	S, DA	SP-only	Built-in tri-axial accelerometer	TBD

[82]	2012	Body Worn	DA, CG	Combined (SP and external accelerometer)	External triaxial accelerometer ADXL345 of Analog Devices connected to a BT-enabled wearable unit	TBD

[62]	2012	Context-Aware	S, DA, CG	SD	Doppler sensor in a Beagle Board-XM embedded computer	PRM: spectral comparison using reference data

[83]	2012	Body Worn	S, DA, CG	SP-only	Built-in tri-axial accelerometer	TBD (five phases)

[84]	2012	Body Worn	S	SP-only	Built-in tri-axial accelerometer and magnetometer	TBD (the decision is externally taken, not decided in the SP)

[85]	2012	Body Worn	CG	Combined (SP with an Arduino Board)	Arduino Duemilanove board with a ADXL335 tri-axial accelerometer and other medical sensors	Presumed TBD

[49,86]	2012	Body Worn	S, DA, CG	SP-only	Built-in tri-axial accelerometer	TBD

[38]	2012	Body Worn	S, DA, CG	SP-only	Built-in tri-axial accelerometer	TBD

[48]	2012	Body Worn	S, DA, CG	SP-only	Built-in tri-axial accelerometer	TBD

[46]	2012	Body Worn	S, DA, CG	Combined (external & internal sensors)	Built-in BMA150 3D accelerometer	Combination of TBD and PRM: Classification Engine that uses a neural network
					External 3-axis MMA7260Q accelerometer (in a Shimmer2 wireless sensor)	

[67]	2012	Body Worn	S, DA	SP-only	Built-in tri-axial accelerometer	PRM: different machine learning classifiers and decision trees.

[87]	2012	Body Worn	S, DA, CG	SP-only	Built-in accelerometer and orientation sensor	Not described

[88]	2012	Body Worn	S, DA, CG	SP-only	Built in accelerometer and orientation sensor	Combination of TBD and PRM (Supervised learning)

[42]	2012	Body Worn	S, DA, CG	SP-only	Built-in tri-axial accelerometer, gyroscope, and magnetic sensor	TBD

[89]	2013	Body Worn	CG	Combined (external sensors)	External Specific Bluetooth-enabled Body Activity Device) with a MXA2500 Dual Axis accelerometer	TBD (mobility detection)

[90]	2013	Body Worn	S, DA, CG	SP-only	Built-in tri-axial accelerometer	TBD

[52]	2013	Body Worn	CG	Combined (external sensors)	External EZ430-Chronos Built-in tri-axial accelerometer	TBD

[91]	2013	Body Worn	S, DA, CG	SP-only (other physiological sensors are included in the system)	Built-in tri-axial accelerometer	TBD

[92]	2013	Body Worn	S, DA	SP-only	Built-in BMA150 3D accelerometer, AK8973 and AK8973 orientation sensor,	PRM: hierarchical rule-based algorithms,

[53]	2013	Body Worn	CG	Combined (external sensor)	TI Sensor Tag with an inertial unit, a barometer, and a temperature and humidity sensor	The detection algorithm is not described

[93]	2013	Body Worn	RMU	SD	Bluetooth-enabled embedded system provided with an accelerometer	TBD

[94]	2013	Body Worn	S, DA, CG	Combined (SP accelerometer and BT medical sensors)	Built-in tri-axial accelerometer (other BT-enabled medical sensors are integrated in the prototype to measures other biosignals)	TBD (combined with the measurement of other vital signals: ECG inspection)

[53]	2013	Body Worn	CG	Combined (external sensor)	TI Sensor Tag with an inertial unit, a barometer, and a temperature and humidity sensor	The detection algorithm is not described

[93]	2013	Body Worn	RMU	SD	Bluetooth-enabled embedded system provided with an accelerometer	TBD

[94]	2013	Body Worn	S, DA, CG	Combined (SP accelerometer and BT medical sensors)	Built-in tri-axial accelerometer (other BT-enabled medical sensors are integrated in the prototype to measures other biosignals)	TBD (combined with the measurement of other vital signals: ECG inspection)

[54]	2013	Body Worn	S, DA	SD (WIMM, Android -based watch)	Built-in tri-axial accelerometer	TBD

[95]	2013	Body Worn	S, DA, CG	SP-only	Built-in tri- tri axial accelerometer and tri axial gyroscope	TBD

[65]	2013	Body Worn and Context-Aware: (bed presence detector)	DA, CG	Combined	BT and ZigBee enabled specific detector (belt) with STM LIS344ALH,	The detection algorithm is not described (decision based on the accelerometry data)
					ZigBee routers in the wall communicate with the SP via BT	

[64]	2013	Combined: Body Worn and Context-Aware system	S, DA, CG	SP-only device combined with external CAS system	Built-in tri-axial accelerometer and external sensors: cameras and microphones for voice recognition and image analysis	TBD

[96]	2013	Body Worn	CG	Combined (SP with an Arduino Board)	Arduino Duemilanove board with a ADXL335 tri-axial accelerometer and other medical sensors	Presumed TBD

[66]	2013	Body Worn	S, DA, CG	SP-only	Built-in accelerometer	TBD

[97]	2013	Body Worn	S, DA	SP-Only	Built-in accelerometer & gyroscope	TBD

[98]	2013	Body Worn	S, DA, CG	SP-only	Built-in accelerometer	TBD

[99]	2013	Body Worn	S, DA, CG	SP-only	built-in tri-axial accelerometer, gyroscope, and magnetic sensor	PRM

[100]	2013	Body Worn	S, DA, CG	Combined (SP accelerometer and BT medical sensors)	Built-in tri-axial accelerometer (other BT-enabled medical sensors are integrated in the prototype to measures other biosignals)	TBD (combined with the measurement of other vital signals: ECG inspection)

[101]	2013	Body Worn	S, DA, CG	SP-only	Built-in tri-axial accelerometer	PRM (Supervised learning)

[35]	2013	Body Worn	S, DA, CG	SP-only	built-in tri-axial accelerometer	TBD

[45]	2013	Body Worn	S, DA, CG	SP-only	Built in accelerometer	TBD

[40]	2013	Body Worn	S, DA, CG	SP-only	built-in tri-axial accelerometer	TBD

[31]	2014	Body Worn	S, DA	SP-only	Built-in tri-axial accelerometer and gyroscope	PRM (decision tree)

[102]	2014	Body Worn	S, CG	SP-only	built-in tri-axial accelerometer	Combination of TBD and PRM

[103]	2014	Body Worn	S, DA, CG	SP-only	Built-in tri-axial accelerometer	TBD

[63]	2014	Combination of Context-Aware & Body Worn	Android sensor Platform (S) SP as a CG	Combined (video data provide the context to interpret activities and reduce false-positives.)	Visual sensors & LilyPad tri-axial accelerometer	PRM (Mann-Whitney test to discriminate activities) and camera data to detect activity.

**Table 2.** Detection performance. Comparative between the different algorithms.

	**Percentage of False Negatives (%)**			**Percentage of False Positives (%)**
	
	**Forward Falls**	**Lateral Falls**	**Backward Falls**	**Other Activities (ADL Movements)**
Basic monitoring of the acceleration [37]	8.0	28.3	5.5	14.6
Fall Index [37]	5.2	13.9	1.8	7.8
*PerFallD* Algorithm	4.5	8.9	14.9	5.9
*iFall* Algorithm	8.0	16.0	12.0	10.1
Brickhouse commercial product [37]	0.8	1.2	29.9	21.9

**Table 3.** Performance of the PerFallD algorithm as a function of the smartphone position.

		**Percentage of False Negatives (%)**			**Percentage of False Positives (%)**
	
		**Forward Falls**	**Lateral Falls**	**Backward Falls**	**Other Activities (ADL Movements)**
*PerFallD*	Waist	4.5	8.9	14.9	5.9
Algorithm	Thigh	3.2	8.7	18.1	20.2
